# Dietary Polysaccharide from *Enteromorpha clathrata* Attenuates Obesity and Increases the Intestinal Abundance of Butyrate-Producing Bacterium, *Eubacterium xylanophilum*, in Mice Fed a High-Fat Diet

**DOI:** 10.3390/polym13193286

**Published:** 2021-09-26

**Authors:** Jiali Wei, Yiran Zhao, Chen Zhou, Qing Zhao, Hongqian Zhong, Xinyu Zhu, Tianyu Fu, Lin Pan, Qingsen Shang, Guangli Yu

**Affiliations:** 1 Key Laboratory of Marine Drugs of Ministry of Education, and Shandong Provincial Key Laboratory of Glycoscience and Glycotechnology, School of Medicine and Pharmacy, Ocean University of China, Qingdao 266003, China; weijiali_gary@163.com (J.W.); zhaoyiran0011@163.com (Y.Z.); zhou_chen2000@126.com (C.Z.); zq010508@163.com (Q.Z.); zhq010607@163.com (H.Z.); zxiny0510@163.com (X.Z.); futianyufty413@163.com (T.F.); pl_panlin@163.com (L.P.); 2 Qingdao Marine Biomedical Research Institute, Qingdao 266071, China; 3 Laboratory for Marine Drugs and Bioproducts, Qingdao National Laboratory for Marine Science and Technology, Qingdao 266003, China

**Keywords:** *Enteromorpha clathrata*, polysaccharide, obesity, *Eubacterium xylanophilum*, gut microbiota, prebiotic, gut dysbiosis, probiotic

## Abstract

Previous studies have suggested that polysaccharide from *Enteromorpha clathrata* (ECP) could be used as a potential prebiotic to treat dysbiosis-associated diseases. However, whether it has any therapeutic effects on obesity has not been investigated. In the present study, we explored the anti-obesity effect of ECP and illustrated that it can significantly reduce the body weight and decrease the serum levels of triacylglycerol and cholesterol in high-fat diet (HFD)-fed mice. As revealed by 16S rRNA high-throughput sequencing and bioinformatic analysis, HFD remarkably changed the composition of the gut microbiota and promoted the growth of opportunistic pathogens such as Mucispirillum, Desulfobacterota and Alphaproteobacteria in obese mice. Interestingly, ECP improved intestinal dysbiosis caused by HFD and reshaped the structure of the gut microbiota in diseased mice by increasing the abundance of butyrate-producing bacterium, *Eubacterium xylanophilum*, in the gut. Altogether, we demonstrate for the first time an anti-obesity effect of ECP and shed new light into its therapeutic mechanisms from the perspective of gut microbiota. Our study will pave the way for the development of ECP as new prebiotic for the treatment of obesity and its associated disorders.

## 1. Introduction

*Enteromorpha clathrate* or *Ulva clathrate*, a marine-derived green alga, has been widely used as a natural herb in Asian countries [1,2,3,4]. Preceding studies have demonstrated a beneficial role of *E. clathrata* for the management of chronic diseases and accumulating evidence has indicated that *E. clathrata* polysaccharide (ECP) is a major bioactive constituent [2,3]. Due to its high molecular weight, ECP is not absorbed after oral intake. As such, when reaching the colon, it could be degraded and fermented by the gut microbiota [5,6]. Fermentation of ECP would change the structure of the gut microbiome and therefore holds great potential for the treatment of dysbiosis-associated diseases [5].

We previously found that ECP could be used as a prebiotic to stimulate the growth of beneficial microbes in the gut including *Bifidobacterium* spp., *Lactobacillus* spp. and *Akkermansia muciniphila* [5]. However, as a novel gut microbiota modulator, whether ECP has any therapeutic effects on obesity, a gut dysbiosis-associated chronic disease, has not been investigated. The prevalence of obesity has been dramatically increased globally and marine algae-derived polysaccharides have been documented to be a good source for the development of anti-obesity agents [7,8,9,10]. For example, fucoidan, a sulfated polysaccharide from brown seaweed, have been illustrated to protect against high-fat diet-induced metabolic syndrome and gut dysbiosis. Specifically, fucoidan could promote the growth of beneficial bacteria, including *Akkermansia muciniphila* and *Alloprevotella* spp. in the gut [9]. Additionally, laminarin, another prebiotic polysaccharide from seaweed could also reduce the body weight in obese mice by favorably modulates the gut microbiota [10]. 

In the present study, we aim to explore the therapeutic effect of ECP on high-fat diet-induced obesity in a mouse model and its beneficial mechanism from the perspective of gut microbiota. Our study will pave the way for the development of ECP as new prebiotic for the treatment of obesity and its associated gut dysbiosis.

## 2. Materials and Methods

### 2.1. Chemicals and Reagents

ECP used in the present study was extracted from the edible green alga *E. clathrata* and prepared using the same method that has been previously described [5]. *E. clathrata* and was obtained from Qingdao Seawin Biotech Group Co., Ltd. (Qingdao, China). The obtained seaweeds were first washed, dried, and then powdered. The lipids and other small molecules in the seaweeds were extracted 3 times with 85% ethanol (Sinopharm Chemical Reagent Co. Ltd., Shanghai, China) at 75 °C for 6h. The resulted seaweeds residues were further treated 3 times with hot water at 90 °C for 6h to obtain ECP. ECP was precipitated using 95% ethanol and was lyophilized after dialysis. The molecular weight, monosaccharide composition and sulfate content of ECP were determined using the protocol described elsewhere [5,9]. ECP has a molecular weight of 11.67 kDa and a sulfate content of 14.7%. ECP is primarily composed of rhamnose (49.7%) and glucose (29.9%). All other chemicals used in the current research were of analytical grade and were obtained from Sigma (Shanghai, China).

### 2.2. Animal Treatment and Sample Collection

The animal experiment in the present study were approved and supported by the Ethical Committee of Ocean University of China, School of Medicine and Pharmacy (Permission No. OUC-2021-0301-01) and complied with the Guide for the Care and Use of Laboratory Animals (National Academies Press, 8th edition, 2011). Briefly, a total of 18 six-week-old male C57BL/6J specific pathogen-free (SPF) mice with an average body weight of about 22 g were purchased from Beijing Vital River Laboratory Animal Technology Co. Ltd. (Beijing, China) (Certificate No. SCXK (Jing) 2016-0011). After a short one-week adaptation period, all mice were randomly allocated into 3 experimental groups (n = 6 mice per group): normal control group (NC), model group (MD) and ECP treatment group (ECP). ECP was dissolved in normal saline and was given by gavage at a dosage of 400 mg/kg/day. The NC and MD group were given an equal volume of normal saline. NC mice were fed with a normal chow diet (D12450B, Research Diets Inc., New Brunswick, NJ, USA). MD mice and ECP mice were fed a HFD (D12492, Research Diets Inc.). 

After about 4 weeks of treatment, all mice were humanely sacrificed. The serum concentrations of triacylglycerol and cholesterol were analyzed and determined using commercially available biochemical kits (Jiancheng, Nanjing, China). The contents in the cecum of each experimental mouse were aseptically collected.

### 2.3. 16S rRNA High-throughput Sequencing and Bioinformatic Analysis of Sequencing Data

The gut microbiota metagenomic DNA was extracted and purified from the cecal contents using a well-established QIAamp-DNA mini-kit for stool samples (Qiagen, Hilden, Germany). After quality checking of the DNA by gel electrophoresis, the hypervariable V3 to V4 regions of the 16S gene were then specifically and efficiently amplified using a pair of widely-used universal primers (338F and 806R) [10,11]. The amplicons were quality-checked and sequenced using an Illumina MiSeq platform (Illumina PE300, San Diego, CA, USA) from Majorbio Bio-pharm Biotechnology Co., Ltd. (Shanghai, China). Raw fastq files data generated from the high-throughput sequencing processes were further analyzed with QIIME pipeline. The obtained sequences and reads were then denoised and the operational taxonomic units (OTUs) were generated by clustering at 97% similarity using UPARSE 7.1. Bioinformatics including α-diversity analysis, clustering and PCA were conducted using the online Majorbio Cloud platform (https://cloud.majorbio.com (accessed on 25 September 2021)) using the protocols previously described [11]. The metabolic functions of the gut microbiota were analyzed and predicted using PICRUSt based on COG database and KEGG database.

### 2.4. Statistical Analysis

Data were expressed as mean ± SEM. Statistical analyses between NC vs. MD and MD vs. ECP were conducted using ANOVA with post-hoc Tukey’s tests (GraphPad Prism 8.00, La Jolla, CA, USA). The linear discriminant analysis (LDA) effect size (LEfSe) analysis with an LDA score of above 3 was performed to visualize and compare the compositional and structure discrepancies between different microbial communities. Pearson’s correlation analysis was used to study the associations between gut microbiota and pathological features of obesity.

## 3. Results and Discussion

### 3.1. Dietary ECP Reduced the Body Weight and Decreased the Serum Levels of Triacylglycerol and Cholesterol in High-Fat Diet (HFD)-Fed Mice

**Figure 1:**
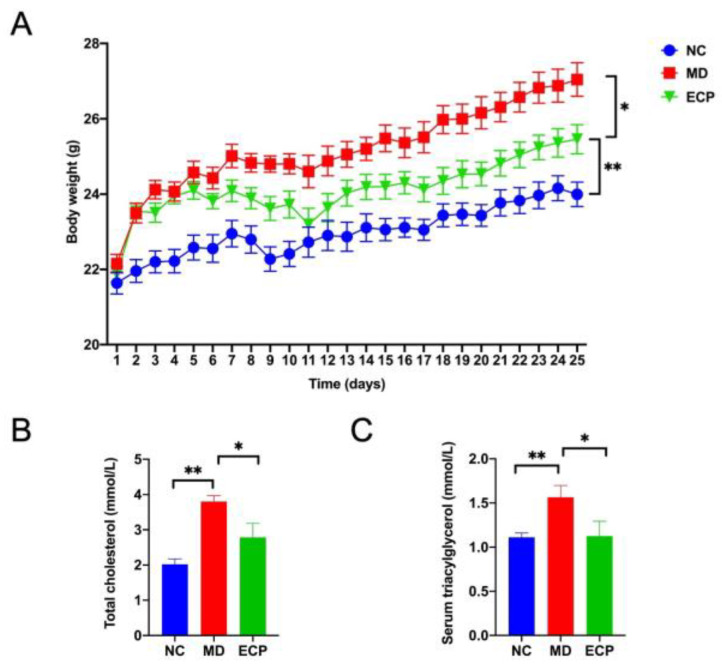
Oral intake of ECP significantly decreased the body weight (**A**), serum levels of triacylglycerol (**B**), cholesterol (**C**) in HFD-fed mice. Data are expressed as means ± standard error of the mean (SEM). * *p* < 0.05, ** *p* < 0.01.

We first explored the anti-obesity effect of ECP using an HFD-induced obesity mouse model. Oral administration of ECP (400 mg/kg) significantly decreased the body weight of HFD-fed mice (Figure 1A). Biochemical analysis indicated that dietary ECP could also re-duce the serum levels of triacylglycerol and cholesterol (Figure 1B,C). Collectively, these results demonstrate a favorable anti-obesity effect of ECP on HFD-fed mice.

### 3.2. Dietary ECP Changed the Overall Structure of the Gut Microbiome in HFD-Fed Mice

**Figure 2:**
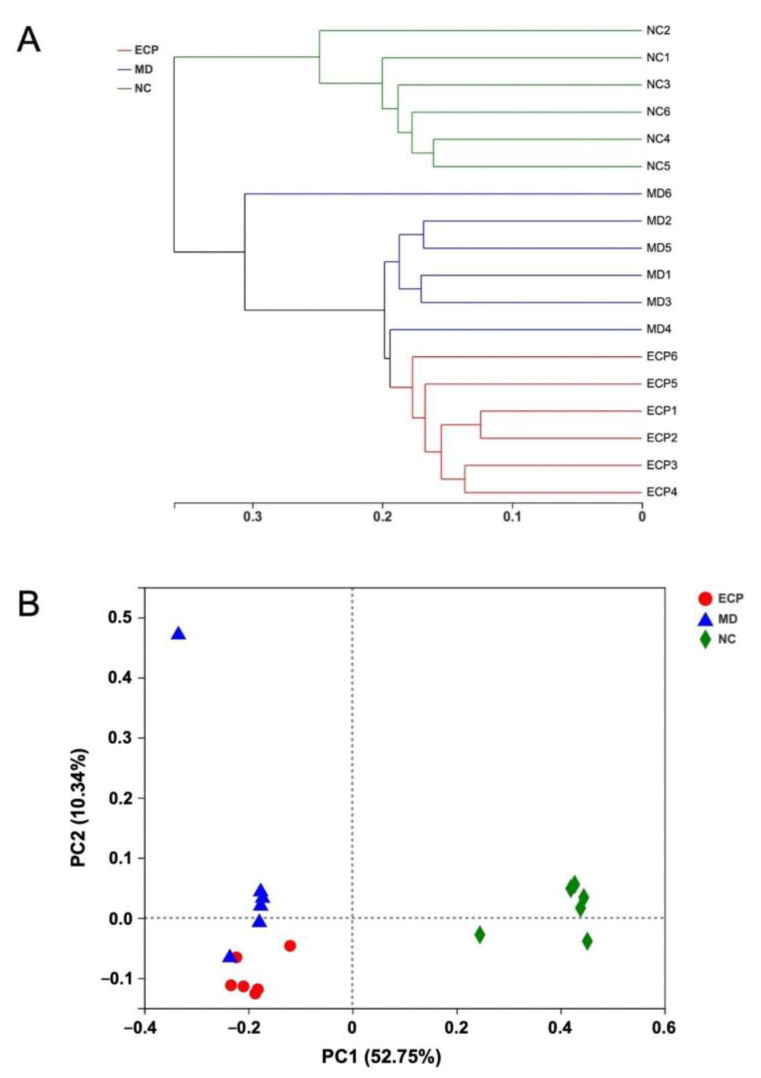
Oral intake of ECP significantly changed the structure of the gut microbiota in HFD-fed mice. Clustering analysis of the gut microbiota (**A**). PCA score plot of gut microbiota (**B**).

Previous studies from our lab indicated that dietary ECP could change the structure of the gut microbiota by increasing the abundance of probiotic bacteria, including *Bifidobacterium* spp., *Lactobacillus* spp. and *A. muciniphila* [5]. HFD-induced obesity is associated with intestinal dysbiosis and preceding studies have illustrated that the gut microbiota is a good target for the management of obesity and metabolic diseases [12,13,14]. In this regard, we further explored the effects of ECP on the gut microbiome. 16S rRNA high-throughput sequencing and bioinformatic analysis indicated that dietary ECP could remarkably change the structure of the gut microbiota in HFD-fed mice (Figure 2A,B). Interestingly, although ECP reshaped the composition of the microbiome, it did not change the α-diversity of the intestinal microbiota (Figure S1).

### 3.3. Dietary ECP Modulated the Composition of the Gut Microbiota at the Phylum and Genus Levels

**Figure 3:**
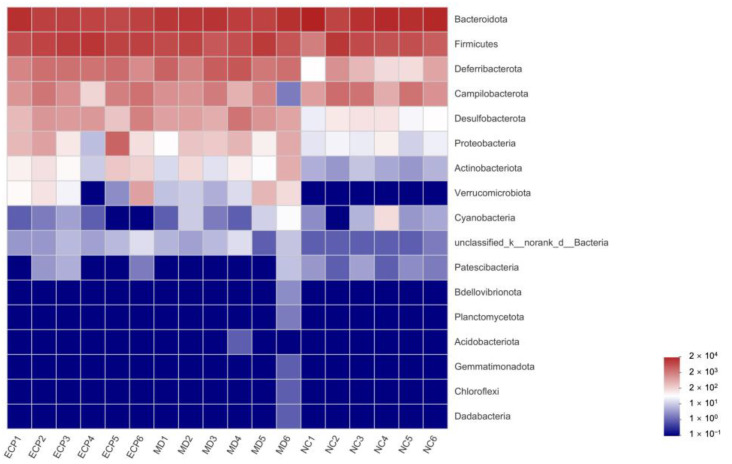
Heatmap analysis of the composition of the gut microbiota at the phylum level. The data are expressed as lg value.

We next investigated the effect of ECP on the gut microbiota at the phylum level. The gut microbiota of the NC mice was dominated by *Bacteroidota* and *Firmicutes* but that of the HFD-fed mice was characterized by *Bacteroidota*, *Firmicutes* and *Deferribacterota* (Figure 3 and Figure S2). Besides, in line with previous results [10,15,16], HFD significantly increased the abundance of dysbiotic bacteria including *Desulfobacterota* and *Proteobacteria* in obese mice (Figure 3 and Figure S2).

**Figure 4:**
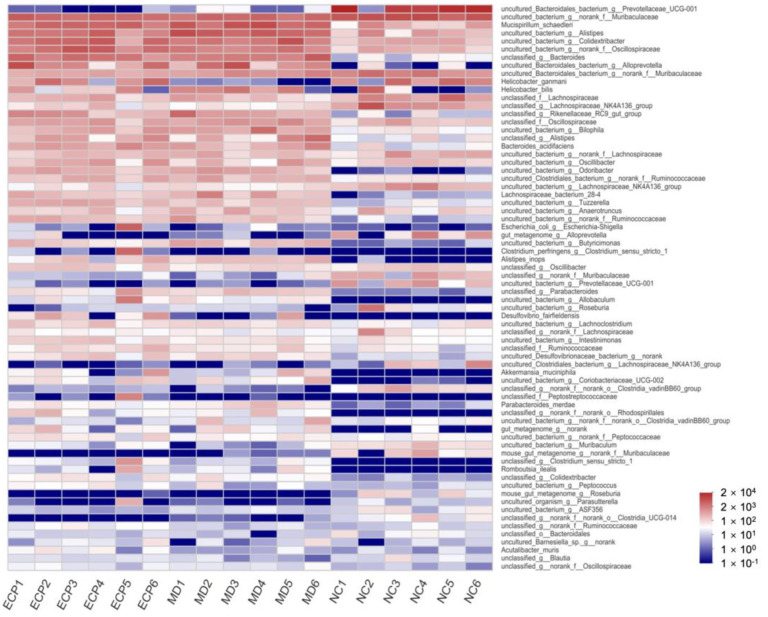
Heatmap analysis of the composition of the gut microbiota at the genus level. The data are expressed as lg value.

We further explored the modulatory effect of ECP on the gut microbiota at the genus level (Figure 4 and Figure S3). Heatmap analysis indicated that HFD and ECP significantly changed the composition of the gut microbiota. The population of *Prevotellaceae UCG-001*, *Butyricimonas*, *Rikenellaceae RC9*, and *Odoribacter* were remarkably altered in response to HFD and ECP treatment.

### 3.4. Dietary ECP Significantly Increased the Intestinal Abundance of E. xylanophilum in HFD-Fed Mice

**Figure 5:**
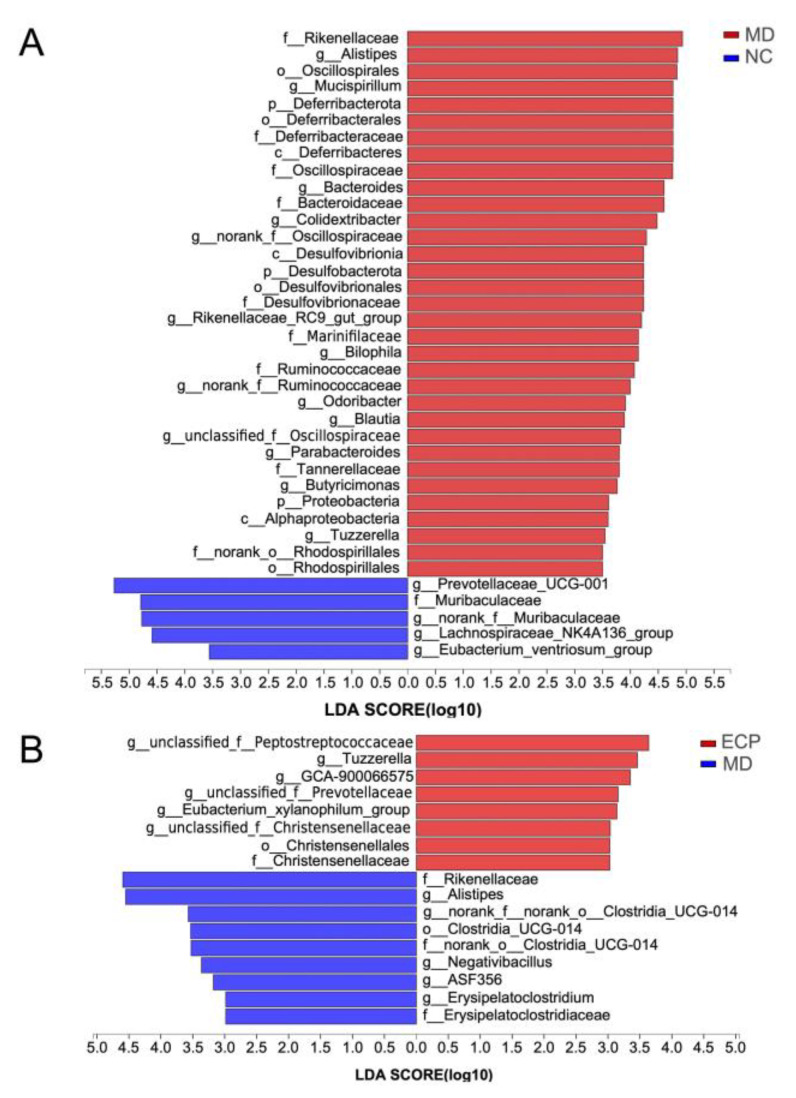
LEfSe analysis of the gut microbiota between NC and MD (**A**) and MD and ECP (**B**) groups.

To investigate the global regulatory effect of ECP on the intestinal microbiome, we then performed the LEfSe analysis. At the genus level, the microbiota of the NC group was primarily dominated by short-chain fatty acid producers, including *Prevotellaceae UCG-001*, *Muribaculaceae*, *Lachnospiraceae NK4A136* and *Eubacterium ventriosum*. These bacteria are critically important for the maintenance of intestinal hemostasis (Figure 5A and Figure S4). In accordance with preceding results, HFD significantly decreased the abundance of beneficial microbes in the gut [10,15,16]. Additionally, HFD promoted the growth of opportunistic pathogens such as *Mucispirillum*, *Alistipes*, *Desulfobacterota* and *Alphaproteobacteria* [10,15,16]. Interestingly, HFD-induced dysbiosis in the gut was reversed by ECP treatment. Oral in-take of ECP significantly increased the population of probiotic bacteria, including *E. xylanophilum*, and *Prevotellaceae* (Figure 5B and Figure S5).

Given that ECP significantly changed the structure of the gut microbiota, we further explored the effect of ECP on the metabolic functions of the gut microbiome (Figure S6A). Clusters of orthologous genes (COG) function analysis indicated that HFD remarkably modified the metabolic capabilities of the gut microbiota including carbohydrate transport and metabolism, energy production and conversion, amino acid transport and metabolism, lipid transport and metabolism and secondary metabolites biosynthesis, transport, and catabolism (Figure S6B). Dietary ECP tended to increase carbohydrate transport and metabolism and decrease energy production and conversion of the gut microbiota but did not reach statistical significance (*p* < 0.05) in this short-term study (Figure S6B).

### 3.5. E. xylanophilum Is Negatively Associated with Body Weight and Serum Levels of Total Cholesterol

**Figure 6:**
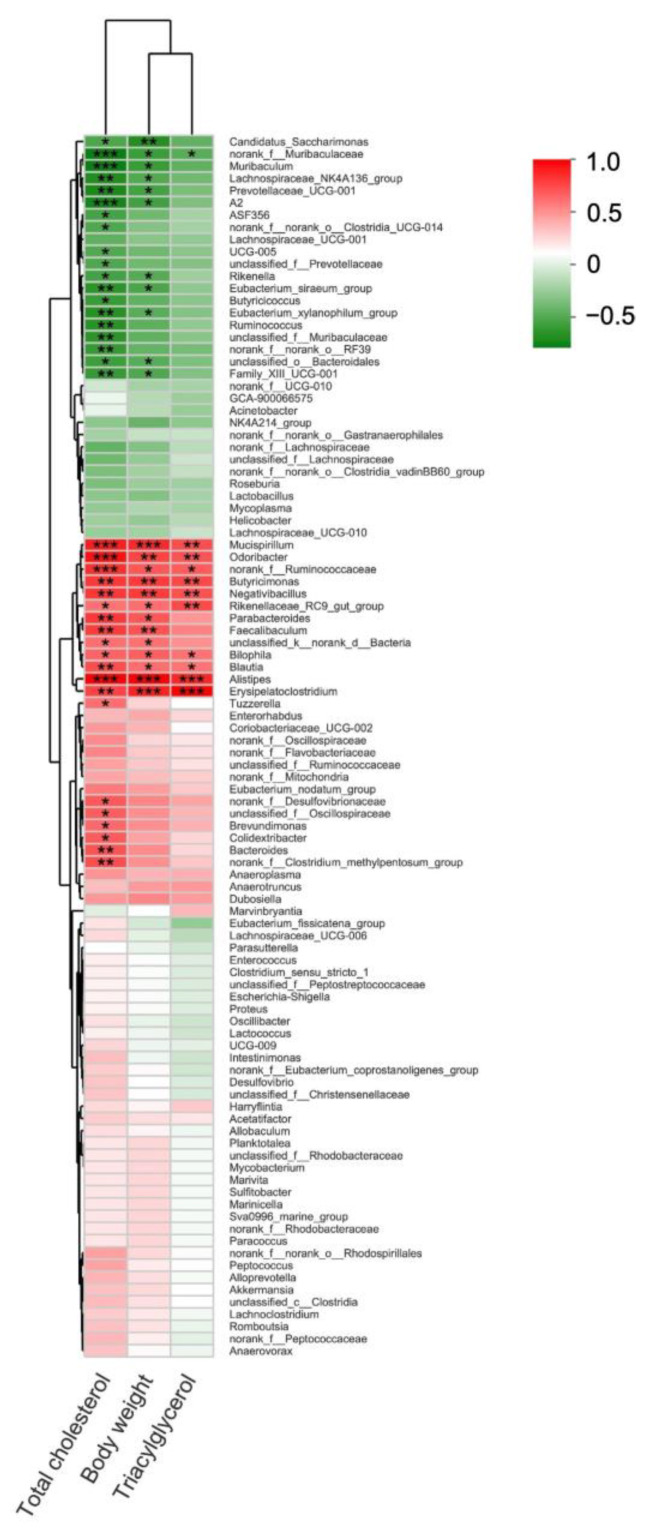
Pearson’s correlation analysis of different gut bacteria at the genus level with triacylglycerol, body weight and cholesterol. Correlations with R > 0.4 or R < −0.4 were identified by asterisks. * *p* < 0.05, ** *p* < 0.01, *** *p* < 0.001.

Since dietary ECP significantly changed the compositions of the gut microbiota in HFD-fed mice at both phylum and genus levels, we then questioned whether or not these changes were associated with improved metabolic parameters of the obese mice. To address this issue, we performed a Pearson’s correlation analysis between gut microbiota and the pathological features of obesity including serum levels of total cholesterol, triacylglycerol, and body weight. In line with previous research, at the phylum level, *Deferribacterota* and *Desulfobacterota* were observed to be positively correlated with body weight and serum lipid levels (Figure S7) [10,15]. Interestingly, at the genus level, we found that *E. xylanophilum* and *Prevotellaceae*, two bacteria that were highly enriched by ECP treatment, were both negatively associated with body weight and serum levels of total cholesterol (Figure 6). 

Short-chain fatty acids (SCFAs), including acetate, propionate, and butyrate, are a class of beneficial fermentation products produced by specific microbes in the gut [17,18,19,20]. Previous studies have demonstrated a beneficial role of SCFAs in the treatment of HFD-induced obesity [21,22,23]. *E. xylanophilum* is a potent butyrate-producing bacterium in the gut and preceding studies have illustrated that polysaccharides from wheat bran could stimulate the production of butyrate of the human microbiota by promoting the growth of *E. xylanophilum* [24,25]. In the present study, we demonstrate for the first time that ECP could attenuate HFD-induced obesity and promote the growth of butyrate-producing bacterium, *E. xylanophilum* in the gut. In light of the fact that *E. xylanophilum* is also negatively associated with body weight and serum levels of total cholesterol, it is therefore possible that ECP could have *E. xylanophilum* as its primary target during attenuation of HFD-induced obesity. However, more detailed studies are warranted to test this possibility. 

In the present study, we primarily focused on elucidating the modulatory effects of ECP on the gut microbiota in HFD-fed mice. Due to the experimental design, relevant in-formation about the food intake of the mice was missed. However, we previously found that dietary ECP could decrease the food intake of healthy mice [5]. Therefore, it is possible that ECP could also reduce the food intake of obese mice fed an HFD. However, further studies are encouraged to explore this possibility and to find out if this is related to the anti-obesity effect of ECP.

Collectively, our study demonstrates for the first time an anti-obesity effect of ECP on HFD-fed mice. Coupled with 16S rRNA high-throughput sequencing and bioinformatic analysis, we further confirmed that the anti-obesity activity of ECP is associated with its modulatory effects on gut microbiota. Specifically, dietary ECP alleviates HFD-induced gut dysbiosis by increasing the abundance of beneficial bacterium, *E. xylanophilum*, in the gut, which is highly relevant for understanding its therapeutic effect.

## 4. Conclusions

In conclusion, we demonstrate an anti-obesity effect of ECP on HFD-fed mice. ECP could significantly reduce the body weight and decrease the serum levels of triacylglycerol and cholesterol in obese mice. Additionally, ECP supple-mentation remarkably improved intestinal dysbiosis in HFD-fed mice by increasing the abundances of probiotic bacteria including *E. xylanophilum* in the gut. Our study will pave the way for the development of ECP as new prebiotic for the treatment of obesity and its associated gut dysbiosis.

## Data Availability

The data presented in this study are available on request from the corresponding author.

## Supplementary Materials

The following are available online at https://www.mdpi.com/article/10.3390/polym13193286/s1, Figure S1: α-diversity analysis of the gut microbiota. Shannon index (A), Ace (B) and Chao index (C)., Figure S2. Gut microbiota composition analysis of the experimental mice at the phylum level. Figure S3. Composition of the gut microbiota at the genus level. Figure S4. LEfSe analysis of the gut microbiota between NC and MD groups. Only taxa with an LDA score of above 3.5 are shown. Figure S5. LEfSe analysis of the gut microbiota between MD and ECP groups. Only taxa with an LDA score of above 3.0 are shown. Figure S6. Clusters of orthologous genes (COG) function analysis of the gut microbiota (A). Different COG functions including carbohydrate transport and metabolism, energy production and conversion, amino acid transport and metabolism, lipid transport and metabolism and secondary metabolites biosynthesis, transport and catabolism were analyzed (B). Different alphabetic characters in panel B indicate significant differences (*p* < 0.05) between groups. Figure S7. Pearson’s correlation analysis of different gut bacteria at the phylum level with triacylglycerol, body weight and cholesterol. Correlations with R > 0.4 or R < −0.4 were identified by asterisks. * *p* < 0.05, ** *p* < 0.01, *** *p* < 0.001.
