# Supplementary material for: Dietary Polysaccharide from Enteromorpha clathrata Attenuates Obesity and Increases the Intestinal Abundance of Butyrate-Producing Bacterium, Eubacterium xylanophilum, in Mice Fed a High-Fat Diet
Source: Polymers (Basel). 2021 Sep 26;13(19):3286. doi: 10.3390/polym13193286 (PMC8512240; doi:10.3390/polym13193286)
Supplement: Supplementary file 1 [file polymers-13-03286-s001.zip › polymers-1376868-supplementary.pdf]

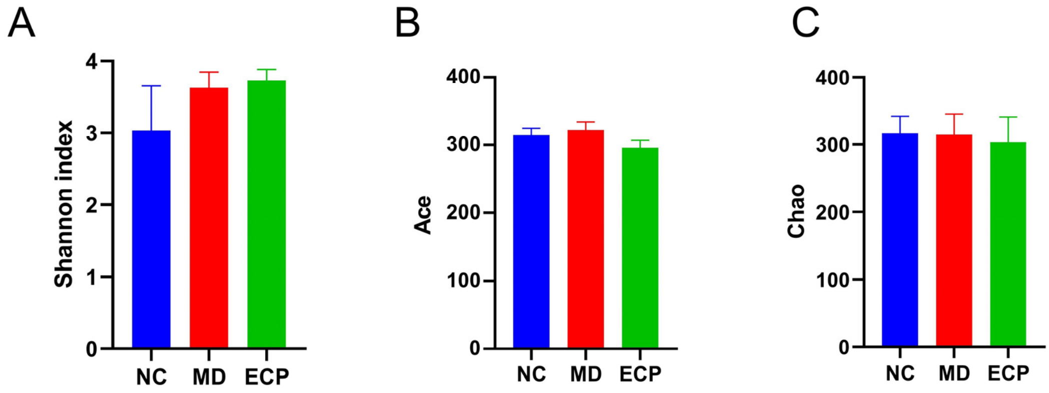

Figure S1. α-diversity analysis of the gut microbiota. Shannon index (A), Ace (B) and Chao index (C).

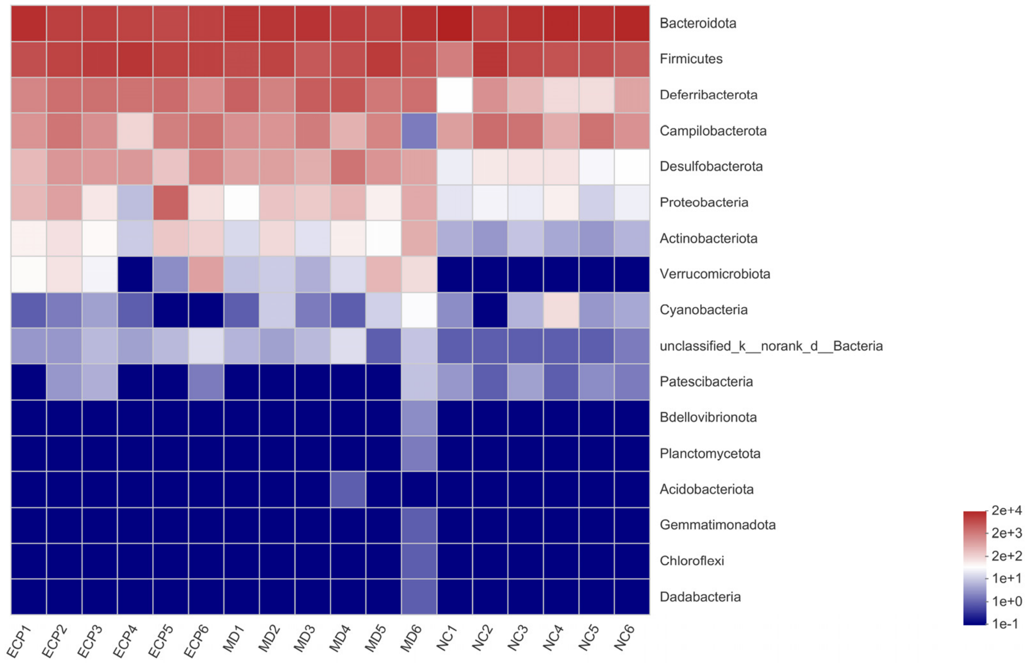

Figure S2. Heatmap analysis of the composition of the gut microbiota at the phylum level.

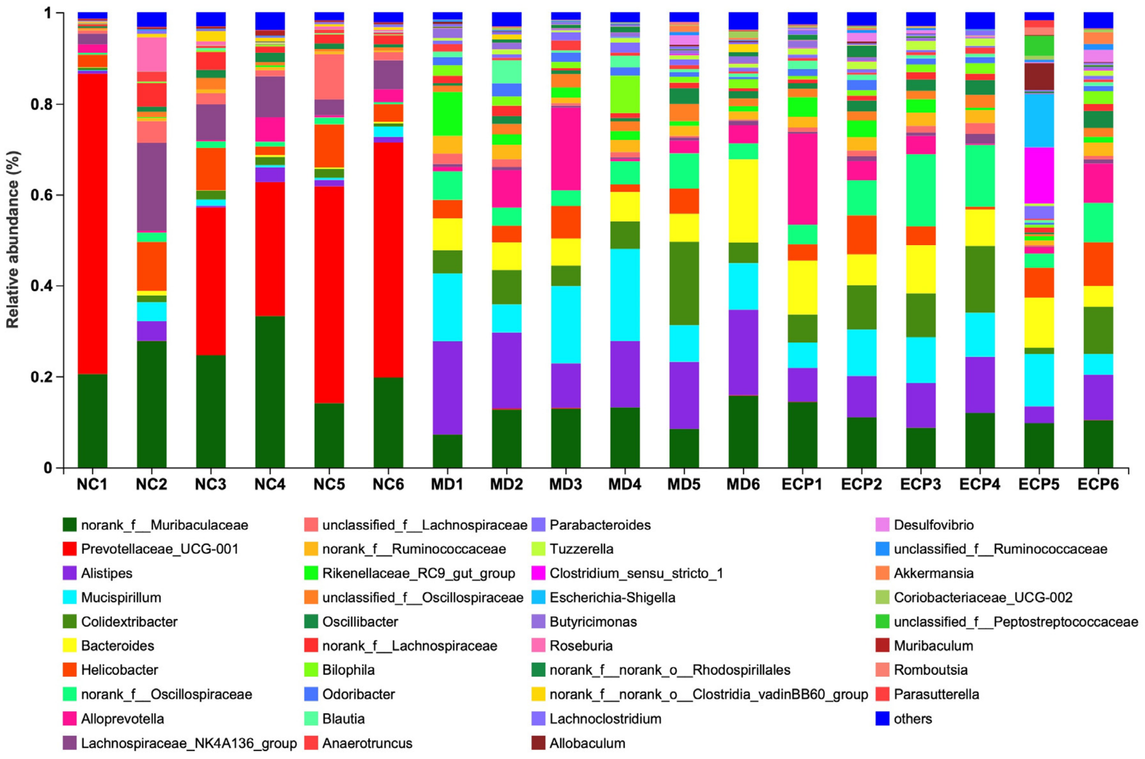

Figure S3. Composition of the gut microbiota at the genus level.

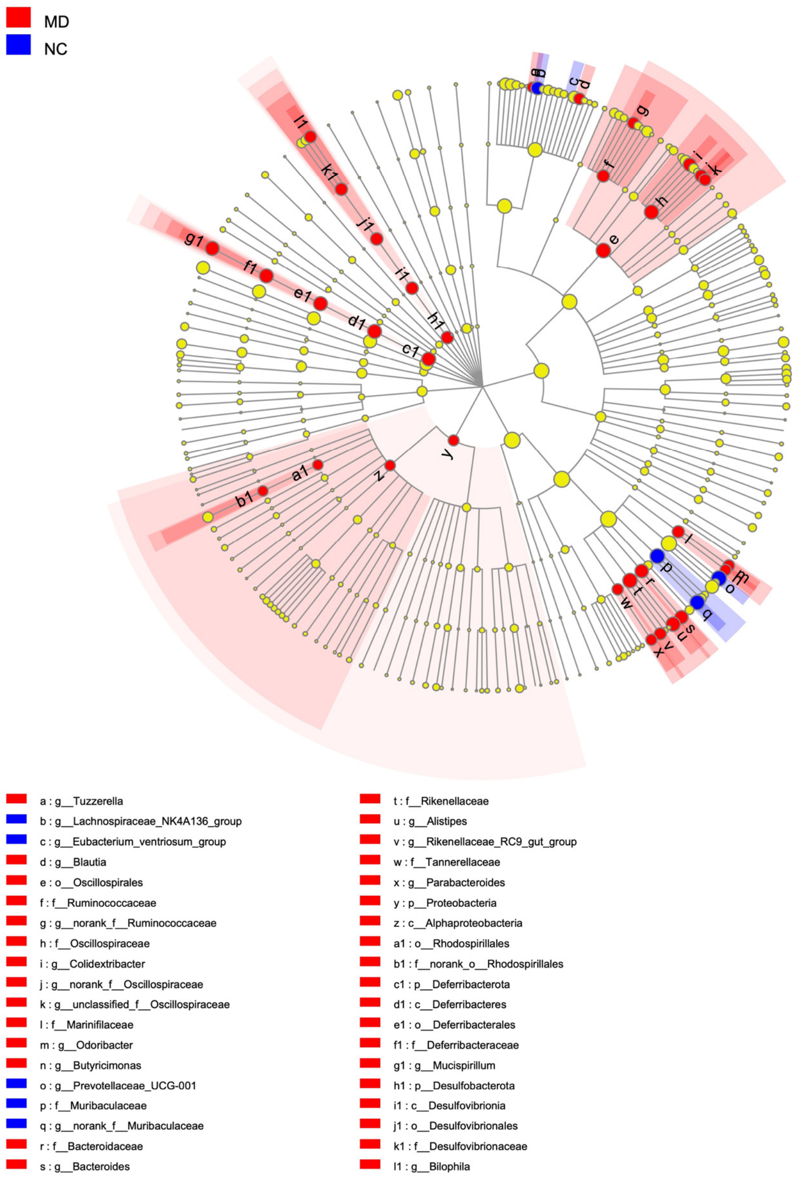

Figure S4. LEfSe analysis of the gut microbiota between NC and MD groups. Only taxa with an LDA score of above 3.5 are shown.

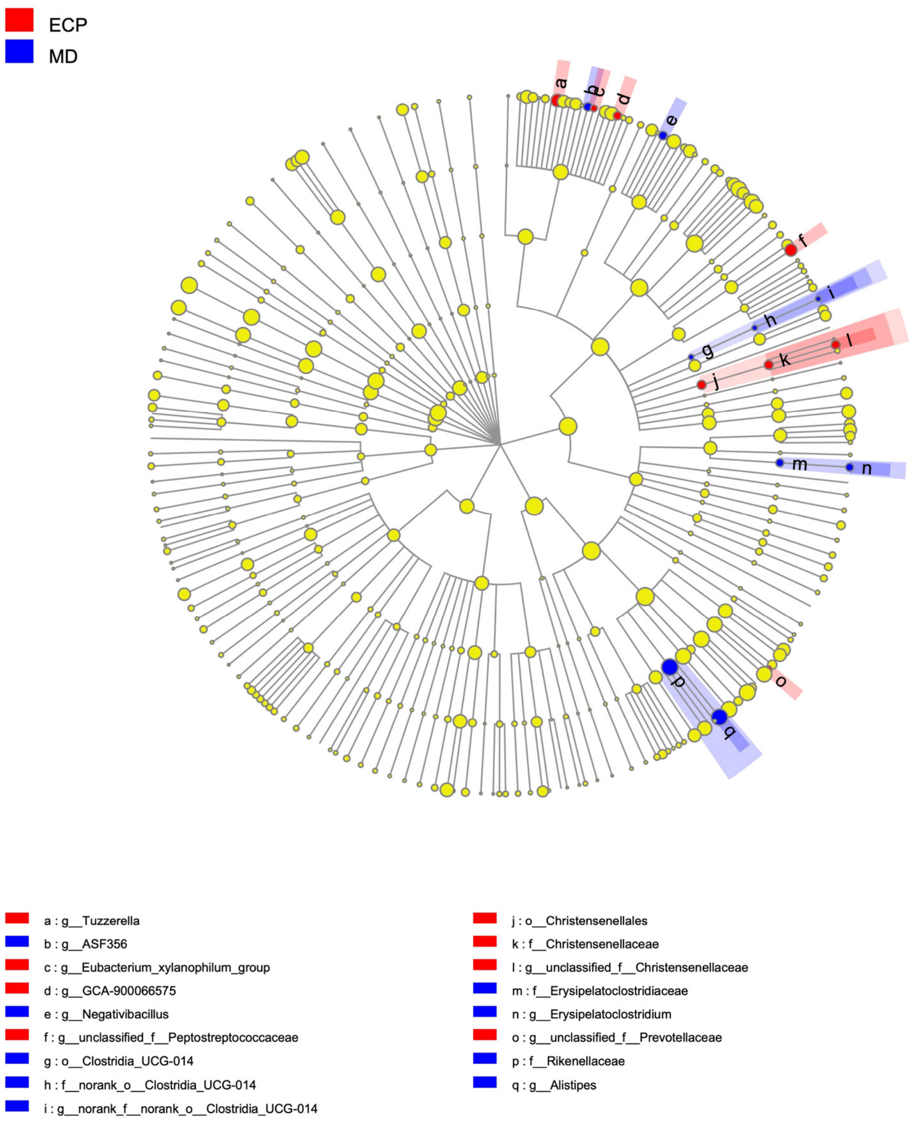

Figure S5. LEfSe analysis of the gut microbiota between MD and ECP groups. Only taxa with an LDA score of above 3.0 are shown.

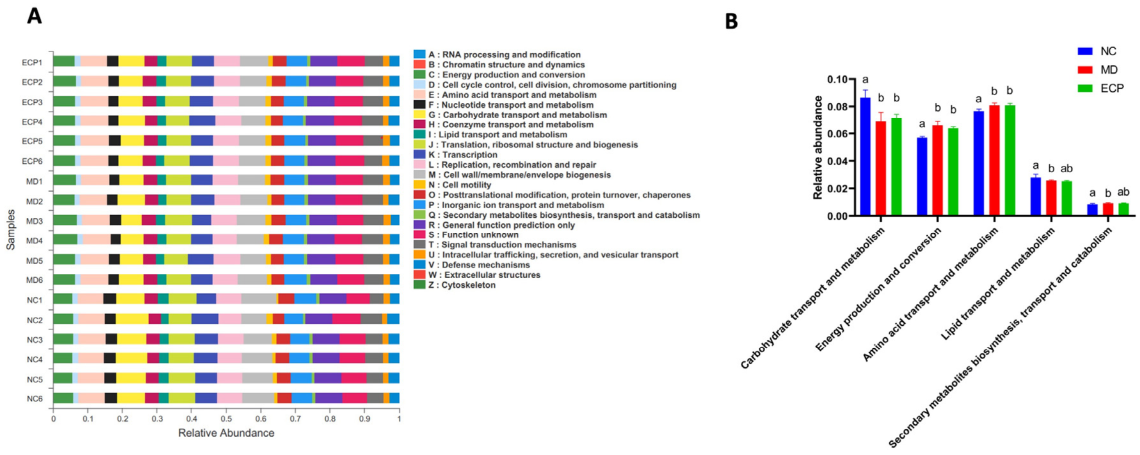

Figure S6. Clusters of orthologous genes (COG) function analysis of the gut microbiota (A). Different COG functions including carbohydrate transport and metabolism, energy production and conversion, amino acid transport and metabolism, lipid transport and metabolism and secondary metabolites biosynthesis, transport and catabolism were analyzed (B). Different alphabetic characters in panel B indicate significant differences ($P < 0.05$) between groups.

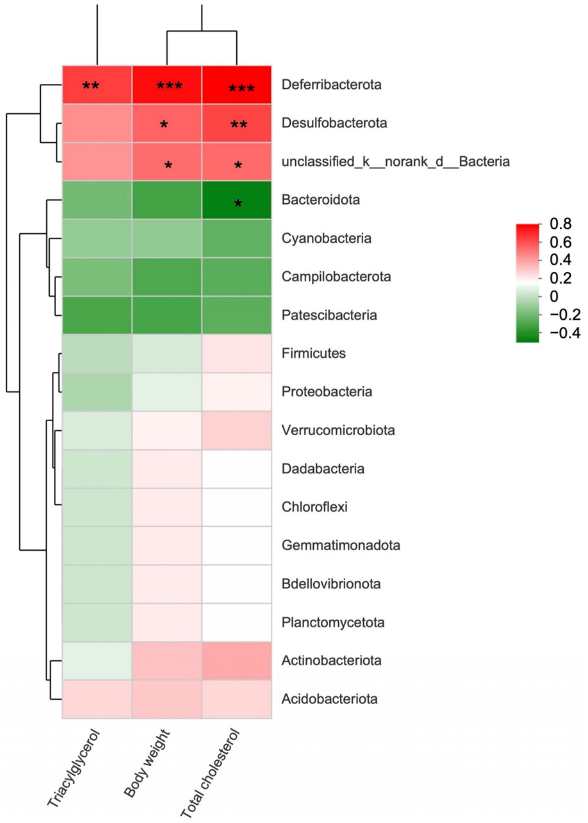

Figure S7. Pearson's correlation analysis of different gut bacteria at the phylum level with triacylglycerol, body weight and cholesterol. Correlations with R > 0.4 or R < −0.4 were identified by asterisks. *P < 0.05, **P < 0.01, ***P < 0.001.
